# Valuing Health and Performance: A Case for Prioritizing Nutrition

**DOI:** 10.1093/milmed/usae522

**Published:** 2024-12-02

**Authors:** Julia Carins, Ben Fisher, Bianka Probert, Joanne L Fallowfield

**Affiliations:** Social Marketing @ Griffith, Griffith Business School, Griffith University, Brisbane, Queensland 4111, Australia; Defence Medical Services, Ministry of Defence, Staffordshire WS14 9PY, UK; Food and Nutrition, Defence Science and Technology Group, Scottsdale, Tasmania 7260, Australia; Institute of Naval Medicine, Ministry of Defence, Alverstoke PO12 2DL, UK

## Abstract

**Introduction:**

Improving the dietary behaviors of personnel can result in positive impact beyond the individual, creating benefits for their organization and wider society. Military personnel endure extended periods of physical and cognitive activity. Healthful dietary behaviors by military personnel support preparedness; yet poor diet behaviors remain common and persistent, and adversely impact health and physical and cognitive performance. Urgent and effective action is needed to improve diet behaviors, but this action has not been prioritized. This study aimed to estimate the value that could be realized from improved diet behaviors to support prioritization of investment in this area for policy and program change.

**Materials and Methods:**

Value estimations (via Social Return on Investment methods) were performed to determine the potential financial benefit derived from improved diet behaviors for 2 military organizations: Australia and the UK. Estimations focused on benefits of reduced attrition and separation, improved productivity, mitigation of musculoskeletal injury (MSKI) risk, and reduced medical claims.

**Results:**

The value of 5 outcomes was estimated for Australia and 3 for the UK. Conservative estimates were of the order of ∼£30 million in the UK (MSKI alone) and ∼$24 million in Australia. These are not insignificant sums of money and could deliver more when invested in health and performance compared with how far they would go toward alleviating attrition, productivity losses, and MSKI.

**Conclusion:**

These estimates were constructed using the best available data and transparency within the calculations, but they remain estimates. The collection of additional data would enable the calculation of further outcomes and increase the usefulness of Social Return on Investment estimation in this area. Militaries should invest greater effort and funding in achieving, maintaining, and optimizing personnel health and performance. Promoting healthy diet behaviors should be prioritized as a cost-effective preventive action that supports productivity and performance, in comparison with the costs of remediating treatment. Conceptualizing the value of improving diet behaviors in monetary terms may refocus efforts on prevention rather than treatment.

## INTRODUCTION

Current military training and operations place unique physiological, cognitive, and emotional demands on personnel.^1^ Service members increasingly work in small teams, and are expected to be more self-reliant, capable, and agile to ensure mission success. This requires high-level preparedness to endure extended periods of activity, with unpredictable rest and recovery, while exposed to various environmental stressors, as well as (potentially) limited access to water and/or food supplies.^1^ Optimal nutritional practices in preparation for deployments should provide the foundation for military health, sustaining normal (physical, cognitive, and emotional) functioning, promoting resilience to stress exposure, while maintaining mission performance.^2^ Diet quality refers to the quantities and variety of foods within the diet (rather than just the nutrient content).^3^ A high-quality diet is aligned with dietary recommendations and delivers optimal nutrient intake, providing the foundation for military health and performance.^4^

There is a wealth of evidence that the volitional diets of personnel away from operational constraints are suboptimal.^5,6^ Poor diet behaviors are generally characterized by low intakes of fruits, vegetables, fiber and wholegrains, and excessive intakes of sugars, fats, and salt.^5–7^ Suboptimal diet behaviors adversely impact physical and cognitive performance, compromise training outcomes, and prolong recovery from illness and musculoskeletal injury (MSKI).^1,8^ Moreover, poor nutritional practices are associated with increasing rates of overweight and obesity in military populations.^9^ Data from the UK Forces indicated that 1 in 4 (27%) personnel were at an increased risk of weight-related poor health,^10^ and in Australia diet quality in personnel has been observed to be low.^11^ Overweight personnel are at greater risk of incurring a MSKI, will take longer to recover, and will likely require more health care resources to return to full fitness. In Australia, 40% of clinical presentations have been attributed to MSKI,^12^ and historical trend data evidence that approximately 10% of the UK military population is medically non-deployable.^13^ There is a need to improve dietary behavior in military populations—by enabling and encouraging consumption of a high-quality diet.^8^

Approaches to addressing poor health in the military have historically focused on individual symptoms (e.g., overweight/obesity), not the causes (e.g., suboptimal diet), where a significant proportion of poor health outcomes are preventable.^14^ Furthermore, educative or behavioral approaches to intervening are common,^3^ which ignore complexities presented by the military context, in both garrison and field settings, such as food provision, serving times, and time to eat, and the food environment/dining location.^7,15^ Moreover, the military context does not consistently encourage the consumption of healthful foods and the maintenance of diets aligned with the nutritional requirements of military roles.^15,16^ Holistic strategies involving comprehensive, systemic change are needed, which requires justification of the associated investment.^17^

Persistent suboptimal dietary behavior and associated ill-health across military populations represent a preventable degrading of human capability at a time when many military organizations worldwide are facing recruitment and retention challenges.^18^ UK recruitment is not keeping pace with workforce outflow,^19^ and Australia continues to recruit below its targeted numbers.^20^ Thus, both are facing declining total force numbers.^20,21^ Both have relatively few levers to influence persons to join the military but can influence the care and retention of personnel through health and performance policies and programs. Retaining personnel for longer capitalizes on their knowledge and expertise, whereas workforce attrition for all reasons represents significant performance and financial liabilities to organizations. Estimating the value that can be realized through intervening to improve the diet behaviors of military personnel could support the prioritization of investment in this area.

Comprehensive calculation of organizational economic costs of the ill-health burden from poor-quality diets, and hence likely cost savings derived from improving diet behaviors, is difficult because of limitations in measuring and reporting health and performance outcome data, and a lack of sufficient budgetary clarity in people-related expenditures. Indeed, an inability to measure the adverse impact of poor-quality diets (at both individual and organizational levels) has probably contributed to the issue not being adequately addressed.

There are methods to determine the value generated from their activities or strategic investments, and some may be suited to this context. Return on investment (ROI) analyses or cost-benefit ratio (CBR) analyses compare the value generated in financial terms (sales) against the investment in producing those sales (operating and capital costs). Early ROI and CBR analyses focussed on tangible costs and financial returns; more recent approaches extended these calculations to less tangible costs, but retained a focus on financial performance.^22^ Therefore, these approaches assume a profit maximization motive; they are not readily applied to public sector organizations (i.e., the military) who do not generate profit *per se*. Health economics evaluates alternative resource allocation models to secure the greatest benefit from health care resources.^23^ Resource allocations (i.e., spending on treatments and/or health system elements) are evaluated in terms of their potential to reduce direct and indirect costs related to illness and disease. Resource costs may include tangible costs, and while disease burden cannot be readily financially determined, it is estimated in monetary terms. Health economic models represent cost reduction approaches, aiming to maximize the efficiency of health care spending in response to ill-health burdens. However, military organizations should seek to maximize productivity and performance by supporting and promoting good health. Social Return on Investment (SROI) determines the value of activities that deliver benefits that are less readily financially determined.^24^ Rather than estimating the ROI from a health intervention as health care costs saved (used to evaluate the effectiveness of a program), SROI estimations focus on forecasting social value—beyond saved health care costs—that could be realized if health-promoting interventions (e.g., behavior change programs) were successfully implemented.^17^ The advantage of the SROI approach is that it provides an estimate in monetary terms for non-tangible benefits that are not easily assigned a value.^24^

There is a need to determine the value that could be realized from intervening to improve the diet behaviors of military personnel. This would support the prioritization of investment in this area for policy and program change. The aim of this paper is to examine two case examples using a SROI-based value estimation approach to calculate potential financial return for (military) organizations from intervening to improve workforce diet behaviors.

## METHODS

### Value Estimation of Improving Dietary Behavior

Value estimations were performed to determine the potential financial benefit that could be derived by improving the diet behavior of personnel in a military context. The estimation focused on the value that would be created if the outcome (improved diet behavior) is achieved. This estimation applied SROI methods,^24^ specifically, the steps involved in identifying desired outcomes: (1) determining indicators for the monetary value of those outcomes, (2) stating assumptions of outcome likelihood, and (3) calculating the combined value of all outcomes.^24^ The estimation was guided by SROI principles,^24^ which include endeavoring to understand outcome changes that are sought or likely and how they are measured, valuing the things that matter, not over-claiming, and providing transparency regarding the information used to establish judgments.

Two case examples were generated from the Australian and UK military organizations to illustrate different applications of SROI to valuing human capability. These case examples were used to compare and contrast the application of the methods and assess their suitability in estimating a value return from behavior change interventions specifically addressing diet behaviors. This research did not involve human participants, and the Griffith University Human Research Ethics Committee determined that it did not require ethical review and approval.

#### Case Example-1; Australian Defence Force

The first case focused on the value that would arise if diet behaviors improved military training course attrition rates, while reducing trainee lost productivity and health care costs. This value estimation also included reducing costs for mid-career personnel separations, as well as flow on costs in terms of reducing veteran medical claims. Public records were used to source trainee participation and attrition rates, career separation rates, and costs of training, health care resources, lost workforce productivity, and veterans’ claims. The degree of change in outcomes that could be expected from improving diet behaviors was estimated from published studies on the efficacy of nutrition interventions in the military setting. To give an example of how these value estimations were calculated, the following calculation was used to estimate the value of a reduction in course failure rate for recruits. The number of recruits who fail training each year (sourced from published records) was multiplied by the cost of training one recruit (sourced from published records) to establish the cost of attrition per year within recruit training. The level of improvement (as a percentage) that may occur when nutrition is improved was conservatively estimated (based on published research). Then the yearly cost of attrition was multiplied by the percentage improvement to arrive at the potential value of improved nutrition within a year (See **Table 1**, row 2). Each estimation is described in the table, with the sources used in the estimation included as footnotes (see supplementary material for a detailed description of the sources used, and justifications for using them). Consistent with SROI principles of full transparency and conservative expectations (i.e., not over-claiming), this estimation likely underestimated the value-return of improved nutrition, as it only focuses on alleviating negative consequences; positive outcomes such as avoidance of poor (physical and mental) health, improved morale, and sustained or enhanced performance are more difficult to account for at this time through assigned monetary value.

**Table 1.** Value Estimation of Improving Dietary Behavior on Trainee Retention, Mid-Career Retention, Mid-Career Health and Productivity, Veteran’s Health (CVD Only)

Personnel	Intended change	Outcome	Indicators	Calculated value	Assumption	Calculation	Potential value
Officer cadets	Trainees perform better during training (reduced course failure rate)	Increased pass rate	160 cadets do not complete training each year (all causes).^a^ The training cost of a cadet (3-year period) was estimated as: $665,072.^b^	$665,072/3 years. *160 cadets is: $35,470,512 per year (attrition costs).	Nutrition interventions may improve pass rate by: 10%.^c^	Yearly costs of: $35,470,512 improvement of: 10%.	$3,547,051
Recruits (all forces)	Trainees perform better during training (reduced course failure rate)	Increased pass rate	357 recruits do not complete training each year (all causes).^a^ The training cost of a recruit (13 weeks + recruiting cost) was estimated as: $55,459.^b^	$55,459 * 357 cadets is: $19,798,846 per year (attrition costs).	Nutrition interventions may improve pass rate by: 10%.^c^	Yearly costs of: $19,798,846 improvement of: 10%.	$1,979,885
Trainees (all forces)	Trainees remain uninjured during training (reduced injury rate)	Reduce health care costs	3871 trainees commence in a year.^a^ The difference in health care costs of obese trainees compared to normal weight trainees over 1 year was estimated as: $4,306.^e^	3871 * $4,306 = $16,667,907 per year (health care costs).	Interventions may decrease BMI by 1 point.^e^ Applied to AUS BMI distribution^f^ reduces OB category by 11%.	Yearly costs of: $16,667,907 improvement of: 11%.	$1,833,470
Trainees (all forces)	Trainees remain uninjured during training (reduced injury rate)	Reduce lost productivity costs	3871 trainees commence in a year^a^. The difference in productivity costs of obese trainees compared to normal weight trainees for 1 year was estimated as: $601.^d^	3871 * $601 = $2,327,013 per year (lost productivity costs).	Interventions may decrease BMI by 1 point.^e^ Applied to AUS BMI distribution^f^ reduces OB category by 11%.	Yearly costs of: $2,327,013 improvement of: 11%.	$255,971
Mid-career personnel	Personnel remain well, during career (reduced separation failure rate)	Reduce separation rate	1466 personnel involuntarily separate in a year (all forces).^a^ Premature separation cost for one person (average all personnel types) was estimated as: $109,786.^b^	1466 * $109,786 = $160,946,478 per year (lost productivity costs).	Nutrition interventions may reduce premature separation by: 10%.^c^	Yearly costs of: $160,946,478 improvement of: 10%.	$16,094,648
Veterans	Veterans have fewer health conditions (fewer health care costs)	Fewer DVA claims	254 successful DVA claims are made in a year for heart disease/hypertension.^g^ The average claim cost is estimated as: $27,785.^g^	254 * $27,785 = $7,057,454 per year (treatment, services, compensation).	Nutrition interventions may decrease heart disease by: 10%.^h^	Yearly costs of: $7,057,454 improvement of: 10%.	$705,745
						Total	$24,416,770

Abbreviations: AUS = Australia; OB = Obesity; DVA = Department of Veterans’ Affairs.

Sources:

a Department of Defence (2019). Women in the ADF report 2017-18. A supplement to the Defence Annual Report 2017-18. https://www.defence.gov.au/annualreports/17-18/Downloads/WomenInTheADFReport2017-18.pdf. Accessed July 1, 2021

b Rudski (2009). The cost of injury to the Australian army. Australian National University; Thesis, with adjustment https://www.rba.gov.au/calculator/annualDecimal.html.

c Farina et al. (2020).^27.^

d Peake et al. (2012)^35^ with adjustment https://www.rba.gov.au/calculator/annualDecimal.html.

e Gravina et al. (2023)^36^; Sackner-Bernstein et al. (2015).^37.^

f AIHW (2022) Overweight and obesity. Retrieved from https://www.aihw.gov.au/reports/australias-health/overweight-and-obesity. Accessed February 22, 2024.

g Westphalen (2019),^38^ with adjustment https://www.rba.gov.au/calculator/annualDecimal.html.

h Bechthold et al. (2019).^39^ See supplementary material for a full description of sources.

#### Case Example-2; UK Ministry of Defence

Scientific evidence supports the relationship between improved diet behaviors of personnel and mitigation of health risks and enhanced military performance. In this second case, estimations focussed on the potential value-return of improving diet behavior with respect to mitigating MSKI, across outcomes of workforce productivity loss (including time spent on rehabilitation or attending medical appointments), health care costs (including health care staff and the overheads of rehabilitation facilities), and the cost of MSKI-related medical discharges. Increased incidence of MSKI and increased use of health care resources once injured are associated outcomes from poor diet behaviors. To give an example of how these value estimations were calculated, the following calculation was used to estimate lost productivity for personnel, capturing the cost of the time they are unable to perform their roles. The number of care pathways is recorded each year (Defence records), and the average number of hours a person spends receiving treatment within a care pathway is known (from practice guidelines). The proportion of personnel that needs additional intensive treatment is also known, as well as the hours involved in that care pathway. These are added together to estimate the total number of hours personnel spend away from duties receiving treatment (total hours of lost productivity). The median salary cost can be obtained to determine an hourly productivity cost. Total hours of lost productivity are multiplied by the hourly productivity cost to establish the cost of lost productivity per year while receiving treatment. The level of improvement (as a percentage) that may occur when nutrition is improved was conservatively estimated (based on published research). Then the yearly cost of lost productivity was multiplied by the percentage improvement to arrive at the potential value of improved nutrition within a year (See **Table 2**, row 1). Again, each estimation is described in the table, with the sources used in the estimation included as footnotes (see supplementary material for a detailed description of the sources used, and justifications for using them). Illustrating the conservative nature of SROI estimation within this example, the median cost of Ordinary Ranks was chosen; this is the lower of the two pay structures but represents the greater proportion of the total capitation costs for the UK Armed Forces—figures that can be obtained from public records. Furthermore, the calculation only estimates the productivity lost within treatment pathways and does not include productivity lost while people are on duty (as perhaps restricted duties). Therefore, this estimation is likely an underestimate of the value-return of improved nutrition, as it only focuses on treatment and other productivity gains, and further positive outcomes, such as sustained or enhanced performance, are not estimated within the Case Example.

**Table 2.** Social ROI, Cost-Savings of Improving Dietary Behavior on MSKI Only

Personnel	Intended change	Outcome	Indicators	Calculated value	Assumption	Calculation	Potential value
All personnel	Personnel remain uninjured during service (reduced incidence of injuries).	Reduce lost productivity costs	30,196 MSKI care pathways^a^ (1-h sessions, 3 per week for 18 weeks = 54 h per person^b^). Total pathways = 1,630,584 h. Approx 10% of injured personnel (3019) attend residential rehabilitation for 3 weeks^c^. 3019 * 3 * 40 h week) = 362,280 h. Total = 1,992,864 h of lost productivity per year. Median cost (capitation rate) of a non-officer salary is £68,604, or £1127 /week or £28/h.^d^	1,992,864 h * £28 = £55,800,136 per year (lost productivity costs)	Improved diet behavior mitigates MSKI impact by: 15%^e^	Yearly costs of: £55,800,136 improvement of: 15%	£8,370,020
All personnel	Personnel remain uninjured during service (reduced incidence of injuries) or use less health care resource when injured.	Reduce health care costs	£35,285,570 cost of Defence rehabilitation personnel, per annum (physiotherapists, exercise rehabilitation instructors, and administration staff).^f^ £339,705 cost of one Medical Officer appointment per injury.^g^ Annual operating costs of national rehabilitation centre (DNRC) £20,155,355.^h^	£35,285,570 + £339,705 + £20,155,355 = £55,780,630 per year (health care costs).	Improved diet behavior mitigates MSKI impact by: 15%^e^	Yearly costs of: £55,780,630 improvement of: 15%.	£8,367,094
All personnel	Personnel remain uninjured during service (reduced medical discharges).	Reduce medical discharges	Average of 749 personnel medically discharged per annum with an estimated pension/lump sum cost of £66,548,000 and £20,124,800 of common law claims.^i^	£66,548,000 + £20,124,800 = £86,672,800 per year (medical discharge costs).	Improved diet behavior mitigates MSKI impact by: 15%^e^	Yearly costs of: £86,672,800 improvement of: 15%	£13,000,920
						Total	£29,738,034

**Sources**:

a UK Defence Statistics (2019). https://assets.publishing.service.gov.uk/media/5d08cd14ed915d42f0b8669a/FOI201906012_-_Regulars_by_MDS_redacted.pdf. Accessed May 10, 2024.

b UK Defence intranet (not dated). Accessed May 10, 2024.

c Care Quality Commission (2021). https://www.cqc.org.uk/sites/default/files/Plymouth_Reional_Rehabilitation_Unit_published_27_August_2021.pdf. Accessed February 24, 2024.

d Parliament UK (2015). Military Manpower Capitation Rates for Financial Year 2015-2016. http://data.parliament.uk/DepositedPapers/Files/DEP2015-0883/Capitation_Rates-Military_Manpower_2015-16-Redacted.pdf. Accessed May 10, 2024 and adjusted per https://commonslibrary.parliament.uk/research-briefings/cbp-9835/.

e Cowan et al. (2011),^40^ Peake et al. (2012),^35^ and Shiozawa et al. (2019).^41.^

f Parliament UK. Ministry of Defence Civilian Manpower Capitation Rates for Financial Year 2015-2016, http://data.parliament.uk/DepositedPapers/Files/DEP2016-0043/Soames-MOD_Civilian_Manpower_Capitation_Rates_2015-16-Redacted-O.pdf. Accessed May 10, 2024. With adjustment per https://www.nhsemployers.org/articles/journey-nhs-pay-over-last-decade and https://www.gov.uk/government/collections/civil-service-pay-guidance. Parliament UK. Ministry of Defence Civilian Manpower Capitation Rates for Financial Year 2015-2016, http://data.parliament.uk/DepositedPapers/Files/DEP2015-0883/Capitation_Rates-Military_Manpower_2015-16-Redacted.pdf with adjustment per https://commonslibrary.parliament.uk/research-briefings/cbp-9835/.

g https://www.bma.org.uk/pay-and-contracts/pay/other-doctors-pay/armed-forces-doctors-pay-scales.

h UK Ministry of Defence (2011) DNRC Feasibility Report. https://www.gov.uk/government/publications/defence-national-rehabilitation-centre-dnrc-feasibility-study-report with adjustment per https://www.bankofengland.co.uk/monetary-policy/inflation/inflation-calculator. Accessed May 10, 2024.

i Defence Statistics (UK) (2021) Report created following request for data. See supplementary material for a full description of sources.

## RESULTS

The value of 5 outcomes to the Australian Department of Defence was estimated (**Table 1**). These outcomes were: a reduction in lost training costs (trainee attrition), reduction in health care costs (through injuries to trainees), reduction in lost productivity (through injuries to trainees), reduced premature separation costs (mid-career separation), and fewer Veteran’s health care claims. The total cost saving from these outcomes is approximately AUD$24M per annum.

In applying SROI principles to UK Defence’s estimated MSKI burden alone (**Table 2**), a very conservative (15%) estimated cost-saving from improving diet quality would be circa £30M per annum. This figure did not include sunk costs (real estate, equipment), utilities (heating, lighting), or headquarter support costs; nor did it include lost productivity because of personnel who were unable to perform parts of their role (as opposed to the entirety of their role).

There are size differences between Australian Defence and UK Defence forces. To enable a reasonable comparison, each value estimate was considered in relation to each country’s total and workforce Defence budgets (**Table 3**). These comparisons indicate that the estimated value realized through improved diet behavior, although different in magnitude, are similar relative to the total and workforce budgets. The comparisons also indicate that funding interventions to improve diet behavior, at cost neutral rates (one dollar/pound invested for each dollar/pound of value expected to be realized), would represent a very small part of the entire total or workforce budget.

**Table 3.** Comparison of Value Estimates for AUS and UK

	Outcomes valued	Value estimate	% of Defence total budget	% of Defence workforce budget
AUS	Trainee retention, mid-career retention, mid-career health and productivity, Veteran’s health (CVD only)	$24,416,770	($24.4 M/$47.0 B^a^), 0.05%	($24.4 M/$14.2 B^a^), 0.17%
UK	Reduction in trainee and service personnel MSKI	£29,738,034	(£29.7 M/£52.8 B^b^), 0.06%	(£29.7 M/£13.8 B^b^), 0.21%

Sources:

a https://www.aspi.org.au/report/cost-defence-aspi-defence-budget-brief-2022-2023. Accessed February 21, 2024.

b https://www.gov.uk/government/statistics/defence-departmental-resources-2023/mod-departmental-resources-2023. Accessed February 21, 2024.

## DISCUSSION

Military leaders recognize people are the foundation of their capability, effectiveness, reputation, strength, and competitive advantage, and should be their number one priority. Prioritizing people means providing leadership, training, equipment—and the organizational culture (accepted norms), means (education, environment, and resources), and opportunity (time) for personnel to enact healthy behaviors. Nutrition is vital for health and military performance,^8^ and enabling personnel to enact nutrition behaviors requires a food offer that meets the nutritional needs of personnel (at the right time, in an environment conducive to consumption); and the provision of engaging and relevant behavioral support to enable personnel to make appropriate choices from the available foods to meet their needs (education and persuasion).^25^ Understanding the potential ROI may influence prioritizing resources and action to deliver these nutrition enablers. Therefore, this paper aimed to provide two case examples of the estimated financial value-return to an organization of intervening to improve the diet behavior of its personnel (i.e., estimated value-return).

To address this aim, this paper estimated a financial value-return for military organizations (as order of magnitude indices) that could arise when the diet behaviors of personnel are improved. The importance of nutrition for military readiness and performance has been known for a long time.^8^ However, it is difficult to provide an estimate of the magnitude of that value to the organization. This paper provides preliminary insights that estimate the financial benefit of improved diet while members are serving (through improved retention of personnel, mitigation of injury risk, and reduction of lost productivity costs), and as costs saving through reduced health care costs among veterans. These estimations were significant, totalling $24.4M for selected outcomes in Australia, and £29.7M for MSKI outcomes in the UK. The $24.4M figure represents 0.05% of the total Australian Defence budget (0.17% of Australian Defence workforce costs), which is relatively similar to the £29.7M which represents 0.06% of the total UK Defence budget (0.21% of UK service and civilian personnel costs).

These estimations do not include all of the value that is likely to arise through improved diet behavior. Other tangible and non-tangible benefits that are expected when personnel regularly consume a nutritious diet include improvements and/or efficiencies within roles through increased physical and/or cognitive performance.^26,27^ Personnel who can maintain or improve job performance may experience increased job satisfaction and improved health and well-being, which contributes to improved retention for the organization. Furthermore, personnel who feel cared for and valued by their organization are likely to benefit at a personal well-being level.^28^ At present, it is difficult to ascribe monetary value to these additional outcomes, but they would certainly be valuable to an organization.

Military organizations should focus on achieving, maintaining, and optimizing health and military performance rather than on remediating action, which means supporting good diet behaviors as a preventive action. Recommendations have been made for a whole system approach around themes of: food provision (a quality food offer to meet health and performance needs and wants), tailoring opening times of dining facilities, shaping the food environment, and training and education.^29–31^ This requires investment (a cost) in facilities and people but the return on this investment can justify these costs, whereas the real-terms costs of inaction may be unaffordable. The cost of making changes cannot be allowed to outweigh the future consequences of not acting,^28^ which would include reduced personnel numbers, reduced productivity within those who remain, and an inability to optimize personnel performance.

When investment is granted, system implementation must be measured and monitored, as “what gets measured gets done,” and ROI must be demonstrated. Measuring and valuing outcomes and impacts will not be straightforward given many are not readily financially determined.^24^ Data are limited to what is available in terms of both costs and outcomes, and to the measures that can be implemented in the near term.^29^ Many military organizations have devolved responsibilities and budgets to smaller divisions, creating less coordination,^31,32^ and less ability to conceptualize, support, and measure systemic action. A whole system approach would require coordination and direction by Leadership but would facilitate and empower many to contribute to systemic change and be accountable for implementing the required actions.^28,31^

The value estimations performed in this paper provide a starting point for extending measurement toward full SROI estimation during implementation of a whole system approach. Measurement in the early stages would require assessing, obtaining, and examining the available data to understand where the data can be “drilled” and where additional measures are required, to fill information gaps, and support the full estimation. The scientific evidence showing the link between diet and health, productivity and performance does not always get the policymakers or leadership to listen.^29,32,33^ Estimation in monetary terms may be an effective way to increase prioritization to benefit personnel and the wider organization.^17^

## LIMITATIONS AND FUTURE RESEARCH

This paper contains limitations that must be acknowledged, and which identify important areas for future research. First, the value estimations of selected outcomes were produced following best practice guidelines (i.e., using the best available data and transparency of estimate calculations); SROI calculations cannot be considered precise—they remain estimates. The development of SROI approaches in this area is needed.^17^ With respect to the best available data, currently, there are few studies that quantify the level of improvement in diet quality following interventions in military populations. Although there is evidence that links diet quality to body mass index (BMI), and BMI to MSKI risk and health care costs at the population level, BMI cannot be considered an accurate proxy for diet quality. Furthermore, these estimates were not a complete estimate of all positive outcomes that were likely to arise once diet behaviors were improved. Routine data collection tends to prioritize adverse outcomes (i.e., illness/injury) and associated health care costs, rather than instances of “good health” (i.e., injury and illness prevention, retention of personnel, and improved performance). Further studies are needed to demonstrate how improving diet quality provides other health and performance benefits (even without BMI change), particularly in military populations. Although we conservatively estimated the contribution of improved diet quality to outcomes based on the evidence available, we acknowledge the multifactorial nature of the outcomes we estimated (MSKI health care burden, attrition, lost productivity, premature separation, and health care claims). Even if the prevalence of good health was known, estimating a financial value is challenging as costs are usually loss-framed (e.g., lost productivity) or associated with remediation expenses (i.e., personnel replacement or expenditure of health resources). Limited quality data is available to support estimations of improved or enhanced productivity or performance,^34^ which prevents the full economic burden of poor-quality food provision—and hence poor diet behaviors—being determined. Research is needed to determine the value of improved performance and productivity by personnel (in either qualitative or quantitative terms), to support estimates in monetary terms. Finally, changing behavior is difficult, which has led to recommendations for systemic approaches to improving diet quality which include both individual and organizational elements.^29–31^ At this time, we do not have full economic cost data for developing and implementing systemic interventions to improve food provision and diet behaviors in the military setting, which would improve SROI calculations in this context.

## CONCLUSION

Systemic change supporting healthy diet behaviors would lead to health and military performance benefits—which is potentially a cost-effective strategy to supporting people objectives. Military organizations have a duty of care to address this, particularly where personnel are geographically isolated, working long and often anti-social hours. This paper estimates the value of improved diet behaviors, as monetized estimates of selected outcomes, where the total and relative value to military organizations was substantial. This provides initial understanding of the value associated with action to improve the diet behaviors of personnel. Further research using this approach is encouraged to provide justification to prioritize action, and then to demonstrate ROI following actions. Until the costs of poor-quality provision and associated poor diet behaviors are fully determined and understood by Senior Military Leaders, the necessary organizational resource and action will not be prioritized.

## Supplementary Material

usae522_Supp

## Data Availability

The data supporting this study’s findings are freely accessible or available upon reasonable request from the corresponding author.

## INSTITUTIONAL CLEARANCE

Institutional clearance approved.
