# Supplementary material for: Valuing Health and Performance: A Case for Prioritizing Nutrition
Source: Mil Med. 2024 Dec 2;190(7-8):e1578–86. doi: 10.1093/milmed/usae522 (PMC12208068; doi:10.1093/milmed/usae522)
Supplement: usae522_Supp [file usae522_supp.zip › usae522_Supp/20240525 VMN Supplementary document.docx]

**Supplementary Information**

**Case Example 1 – Detailed justification of claims.**

**1. 160 cadets do not complete training each year (all causes); 357 recruits do not complete training each year (all causes).** Attrition rates are reported in Annex B of the report listed below.

Sources: ^a^ Attrition rates: Department of Defence (2019). Women in the ADF report 2017–18. A supplement to the Defence Annual Report 2017–18. <https://www.defence.gov.au/annualreports/17-18/Downloads/WomenInTheADFReport2017-18.pdf>. Accessed 1 July 2021;

**2. The training cost of a cadet (3-year period) was estimated as $665,072; The training cost of a recruit (13 weeks + recruiting cost) was estimated as $55,459.** Training costs are reported in a published report, but recent costs are not available. Costs were adjusted to current-day rates using the Australian national inflation calculator.

**Sources:** ^b^ Rudski (2009) The cost of injury to the Australian army. Australian National University; Thesis. Available at: <https://www.pc.gov.au/__data/assets/pdf_file/0018/229032/sub040-veterans-attachmentb.pdf>. Accessed 25 May 2024. Adjusted using <https://www.rba.gov.au/calculator/annualDecimal.html>. Accessed 25 May 2024.

**3. Nutrition interventions may improve pass rate by 10%.** Conservative estimate based on published research that shows a 13% improvement in successful course selection between the least and most healthful quartiles of eaters (reported in Table 3 of the paper).

**Source:** ^c^ Farina EK, Thompson LA, Knapik JJ, Pasiakos SM, Lieberman HR, McClung JP. Diet quality is associated with physical performance and special forces selection. *Medicine and Science in Sports and Exercise.* 2020;52(1):178.

**4. 3871 trainees commence in a year.** Commencement numbers are reported in Annex B of the report listed below.

**Source:** ^a^ Commencement numbers: Department of Defence (2019). Women in the ADF report 2017–18. A supplement to the Defence Annual Report 2017–18. [https://www.defence.gov.au/annualreports/17-18/Downloads/WomenInTheADFReport2017-18.pdf. Accessed 1 July 2021](https://www.defence.gov.au/annualreports/17-18/Downloads/WomenInTheADFReport2017-18.pdf.%20Accessed%201%20July%202021)

**5. The difference in healthcare costs of obese trainees compared to normal weight trainees over one year estimated as $4,306.** Healthcare/Productivity costs are reported in published research, recent costs are not available. Costs were adjusted to current-day rates using the Australian national inflation calculator.

**Source:** ^d^ Peake J, Gargett S, Waller M, et al. The health and cost implications of high body mass index in Australian defence force personnel. *BMC Public Health*. 2012;12:451. Adjusted using <https://www.rba.gov.au/calculator/annualDecimal.html>. Accessed 25 May 2024.

**6. Interventions decreased BMI by 1 point.** Conservative estimate of BMI reduction (of 1 BMI point) from interventions based on 0.3 points reduction (Gravina et al., 2023); and 2.8 points reduction (Sackner-Bernstein et al., 2015).

**Sources:** ^e^ Gravina D, Keeler JL, Akkese MN, et al. Randomized Controlled Trials to Treat Obesity in Military Populations: A Systematic Review and Meta-Analysis. *Nutrients.* 2023;15(22):4778; and Sackner-Bernstein J, Kanter D, Kaul S. Dietary intervention for overweight and obese adults: comparison of low-carbohydrate and low-fat diets. A meta-analysis. *PloS one.* 2015;10(10):e0139817l.

**7. Applied to AUS BMI distribution reduces OB category by 11%.** Application of a BMI reduction of 1 point was applied to the distribution of BMI among persons aged 18 and over in Australia, and the percentage reduction in the category calculated from this.

**Source:** ^f^ Distribution of BMI among persons aged 18 and over. AIHW (2022) Overweight and obesity. Retrieved from <https://www.aihw.gov.au/reports/australias-health/overweight-and-obesity>. Accessed 22 February 2024.

**8. 254 successful DVA claims are made in a year for heart disease / hypertension.** Claim rates have been published in the source below.

**Sources:** ^g^ Westphalen N. Compensation in the Australian defence force. *Journal of Military and Veterans Health.* 2019;27(1).

**9 The average claim cost is estimated as $27,785.** Compensation rates have been published, but recent costs are not available. Costs were adjusted to current-day rates using the Australian national inflation calculator.

**Sources:** ^g^ Westphalen N. Compensation in the Australian defence force. *Journal of Military and Veterans Health.* 2019;27(1). Adjusted using <https://www.rba.gov.au/calculator/annualDecimal.html>;

**10 Nutrition interventions may decrease heart disease by 10%.** A conservation estimate of risk reduction was made based on observed risk reduction of 5%-33% risk reduction due to increased or decreased consumption in certain food groups.

**Source:** ^h^ Bechthold A, Boeing H, Schwedhelm C, et al. Food groups and risk of coronary heart disease, stroke and heart failure: a systematic review and dose-response meta-analysis of prospective studies. *Critical reviews in food science and nutrition.* 2019;59(7):1071-1090.

**Case Example 2 - Detailed justification of claims.**

**1. 30,196 episodes of MSKI care pathways were recorded in 2019, and the average care pathway was 18 weeks.** The statistic is reported in the following source.

**Sources:** ^a^ Freedom of Information Request: UK Defence Statistics (2019) The number of UK regular armed forces personnel at 1 April 2019 by medical deployability standard (MDS) and service. Available at: <https://assets.publishing.service.gov.uk/media/5d08cd14ed915d42f0b8669a/FOI201906012_-_Regulars_by_MDS_redacted.pdf>. Accessed 10 May 2024.

**2. The average care pathway is 54 hours per person.** This estimate is based on 3 one-hour rehabilitation sessions per week, as recommended in best practice guidelines for rehabilitation, projected across the 18 weeks care pathway.

**Sources:** ^b^ Defence Rehabilitation, Best Practice Guidelines: UK Defence intranet (not dated). Accessed 10 May 2024

**3. Approximately 3019 personnel attend a 3-week residential rehabilitation course.** Approximately 10% of injured personnel attend a residential rehabilitation course. 10% of 30,196 episodes is 3019 personnel. Three weeks is 120 hours, based on a 40-hour week.

**Sources:** ^c^ Estimated from 14 Regional Rehabilitation Units delivering 2 courses a month of 15 people each. Care Quality Commission, 2021 Inspection Report - Plymouth Regional Rehabilitation Unit. <https://www.cqc.org.uk/sites/default/files/Plymouth_Reional_Rehabilitation_Unit_published_27_August_2021.pdf>. Accessed 24 February 2024

**4. The median cost to Defence (capitation rate) of a non-officer salary is £68,604, or £1127 /week or £28 /hour.** The rank of OR3 was used, which is likely an underestimate given the rank profile in the UK armed forces (Armed Forces’ Pay Review Body: Fifty-Second Report 2023, page 18). The MoD capitation costs for service personnel (which include salary, pension, and national insurance costs) are publicly available for 2016 but not more recently. The published annual pay rise percentages for service personnel for 2016-2023 were applied to the 2011 capitation figure.

**Sources:** ^d^ Parliament UK. Military Manpower Capitation Rates for Financial Year 2015-2016 (page B-1). <http://data.parliament.uk/DepositedPapers/Files/DEP2015-0883/Capitation_Rates-Military_Manpower_2015-16-Redacted.pdf>. Accessed 10 May 2024. Pay costs updated to 2023 as per <https://commonslibrary.parliament.uk/research-briefings/cbp-9835/>, Armed Forces’ Pay Review Body: Fifty-Second Report 2023 (page 18): <https://assets.publishing.service.gov.uk/media/64affa97c033c1000d806281/AFPRB_2023_Accessible.pdf>

**5. Improved diet behaviour mitigates MSKI impact by 15%.**

Conservative estimate of 15% utilised, reflecting on Cowan et al. (2011) who demonstrated that overweight personnel had 47% increased incidence of MSKI and 49% higher health care utilisation; Peake et al (2012) who demonstrated >20% increased healthcare resource use in overweight/obese military cohorts; and Shiozawa et al (2019) who demonstrated overweight service personnel had 15% more medical appointments.

**Sources:** ^e^ Cowan D, Bedno S, Urban N, Yi B, Niebuhr D. Musculoskeletal injuries among overweight army trainees: incidence and health care utilization. *Occupational Medicine*. 2011;61(4):247-252; Peake J, Gargett S, Waller M, et al. The health and cost implications of high body mass index in Australian defence force personnel. *BMC Public Health.* 2012;12:451; Shiozawa B, Madsen C, Banaag A, Patel A, Koehlmoos T. Body mass index effect on health service utilization among active duty male United States Army soldiers. *Military Medicine.* 2019;184(9-10):447-453.

**6. £35,285,570 cost of Defence rehabilitation personnel, per annum (physiotherapists, exercise rehabilitation instructors and administration staff).** The MoD capitation costs for civilian personnel (which include salary, pension and national insurance costs) are publicly available for 2016 but not more recently. Therefore, the published annual pay rise percentages for 2016-2023 were applied to the 2011 capitation figure. There are three employment groups (i.e., service personnel, NHS ‘agenda for change’ staff (e.g., physiotherapists and ERI, and civil service admin staff). Therefore, different source documents were utilised for each group for the annual percentage pay increases to calculate the 2023 estimate from the 2016 capitation cost. Physiotherapists (civilian): Approximately 300 civilian physiotherapists at Band 6 capitation cost £51,571; Physiotherapists (military): Approximately 120 military physiotherapists at Capt rank/OF2 capitation cost £85,016; ERI: approximately 200 ERIs at average Band-5 capitation cost £42,104; Administrative staff: approximately 50 admin at E2 grade at £23,831. Note: Band 6 and Capt/ OF2 grade selected, however likely to under-estimate costs as does not include the £ premia for Maj/OF3 and Band 7 physiotherapists.

**Sources:** ^f^ Civilian staff (physio, ERI, admin): Capitation costs from Parliament UK. Ministry of Defence Civilian Manpower Capitation Rates for Financial Year 2015-2016: <http://data.parliament.uk/DepositedPapers/Files/DEP2016-0043/Soames-MOD_Civilian_Manpower_Capitation_Rates_2015-16-Redacted-O.pdf> (admin grade, page B-1, physio page B-2). Pay costs updated to 2023 as per: (1) <https://www.nhsemployers.org/articles/journey-nhs-pay-over-last-decade> (2) <https://www.gov.uk/government/collections/civil-service-pay-guidance>. Military physiotherapists: Military capitation costs from Parliament UK. Ministry of Defence Civilian Manpower Capitation Rates for Financial Year 2015-2016 (page B-1; Army Pay, Section B3, Main Regular Officers, Nursing Officers, Captain OF2). <http://data.parliament.uk/DepositedPapers/Files/DEP2015-0883/Capitation_Rates-Military_Manpower_2015-16-Redacted.pdf>. Pay costs updated to 2023 as per: <https://commonslibrary.parliament.uk/research-briefings/cbp-9835/>

**7 £339,705 cost of one Medical Officer appointment per injury.** Estimated hourly cost of a Medical Officer derived from Medical officer 2023 salary of £83409 (divided by 46 weeks, then divided by 40 hours per working week)= £45. Then assumes one Medical Officer appointment per patient and 15 minutes total appointment time, including admin. (£45 an hour/4*30,196 patients). This is the Medical officer's salary only and does not include the cost of other staff intermittently involved (i.e. nurses, podiatrists, pharmacists, occupational therapists, occupational health doctors, and mental health practitioners). This recent salary data for military doctors doesn’t include pension and national insurance costs, so it is likely an underestimate.

**Source:** ^g^ <https://www.bma.org.uk/pay-and-contracts/pay/other-doctors-pay/armed-forces-doctors-pay-scales> page 119.

**8. Annual operating costs of national rehabilitation centre (DNRC) £20,155,355.** The running costs of the national rehabilitation centre are £30,538,418, as reported in the source document. Adjusted to 2023 £ using inflation calculator. Further reduced by one-third to account for (a) healthcare salaries already included in claim 6 above and (b) to account for non-MSKI patients treated at DNRC.

**Sources:** ^h^ UK Ministry of Defence (2011) DNRC Feasibility Report, p61: [https://www.gov.uk/government/publications/defence-national-rehabilitation-centre-dnrc-feasibility-study-report Accessed 10 May 2024](https://www.gov.uk/government/publications/defence-national-rehabilitation-centre-dnrc-feasibility-study-report%20Accessed%2010%20May%202024); adjusted using <https://www.bankofengland.co.uk/monetary-policy/inflation/inflation-calculator>

**9. Average of 749 personnel medically discharged per annum with an estimated pension / lump sum cost of £66,548,000 and £20,124,800 of common law claims.**

**Source**: ^i^ Discharge Statistics 2021, report created following a request for data from Defence Statistics Health & Digital Head, Defence Statistics, MOD Abbey Wood North, Bristol, United Kingdom. Available on reasonable request.
